# Utilization of Complementary and Alternative Therapies in Youth with Developmental Disabilities

**DOI:** 10.1155/2019/3630509

**Published:** 2019-06-25

**Authors:** Rachel Tenenbaum, Rumi Agarwal, Marcus S. Cooke, Mavara M. Agrawal, Marlaina Maddux, Shanna L. Burke

**Affiliations:** ^1^Department of Psychology, Florida International University, Miami, FL, USA; ^2^Robert Stempel College of Public Health and Social Work, Department of Health Promotion and Disease Prevention, Florida International University, Miami, FL, USA; ^3^Oxidative Stress Group, Department of Environmental Health Sciences and Biomolecular Sciences Institute, Florida International University, Miami, FL, USA; ^4^Department of Humanities, Health and Society, Herbert Wertheim College of Medicine, FIU Embrace Initiative, Florida International University, Miami, FL, USA; ^5^Easterseals Blake Foundation, Tucson, AZ, USA; ^6^Robert Stempel College of Public Health and Social Work, School of Social Work, Florida International University, 11200 SW 8th St., AHC5 585, Miami, FL 33199, USA

## Abstract

Oxidative stress is understood to be involved in the ontology and maintenance of different developmental disabilities. Some complementary and alternative medicine (CAM) therapies have been proposed to modify this relationship by affecting oxidative stress pathways. However, it is unclear which of these CAM therapies are used among children with different developmental disabilities. This study examines the use of these therapies among 10,218 children between the ages of 4 and 17 using the 2012 Child Complementary and Alternative Medicine (CAM) Supplement of the National Health Interview Survey (NHIS) to highlight a potential avenue for intervention and prevention efforts. The results suggest that children with developmental disabilities are more likely to utilize particular CAM therapies that may alter oxidative stress pathways. Future work is needed to assess the potential moderating effect of these CAM therapies and oxidative stress levels among children with different developmental disabilities.

## 1. Introduction

The term developmental disabilities encompasses a heterogeneous group of conditions that arise in early childhood and are characterized by difficulties across different domains of functioning and include autism spectrum disorders (ASD), intellectual disability (ID), cerebral palsy (CP), Down syndrome (DS), and other developmental delays (DD) [1, 2]. The National Health Interview Survey (NHIS) of 2016 estimated 6.99% of children were diagnosed with select developmental disabilities (ASD, ID, and DD) based on parent report, an increase from 5.76% based on the 2014 NHIS (Zablotsky, Black, & Blumberg, 2017). More recent data, estimates a prevalence of 3.2 of 1000 children (ages 3-17) (95% CI: 2.7, 3.7) with cerebral palsy, and 11.1 per 1000 children (95% CI: 10.2, 12.1) diagnosed with intellectual disability [3]. Furthermore, the presence of developmental disabilities is often associated with functional limitations and youth with developmental disabilities often require varying degrees of life-long care. Support and research are needed to examine prevention and intervention efforts for these conditions [4]. However, the etiology of these conditions is poorly understood and the need to elucidate the biological basis of these conditions is of utmost importance in order to properly inform prevention and intervention targets [5].

One emerging body of literature implicates a role for oxidative stress in the development and maintenance of these conditions, which may serve as a potential arena for prevention and intervention efforts. Oxidative stress originates from the overproduction of reactive oxygen species (ROS), arising from multiple exogenous and endogenous sources, including inflammation, the diminution of antioxidant defenses, or a combination of both [6, 7]. Given the vulnerability of the brain to oxidatively generated damage, oxidative stress has been implicated in the pathogenesis of various psychiatric and medical conditions [8]. Furthermore, there is a growing body of literature to suggest that oxidative stress and the resulting damage to cellular molecules have a mechanistic role in aspects of developmental disabilities, given the potential neurodegenerative changes associated with the ontology of these conditions [8]. Specifically, a vulnerability to neurodegeneration occurs due to a lack of ability to combat oxidative stress in the brain despite a large oxidative capacity with multiple sources of ROS, and oxidative stress-derived damage to cellular biomolecules can result in neuronal dysfunction and brain tissue loss [9–15].

While the literature is evolving regarding the relationship between oxidative stress and developmental disabilities, the role that oxidative stress plays in the reduction of symptoms or impairment has yet to be examined. Specifically, research examining the use of particular therapies to decrease oxidative stress in this population is in its infancy. However, investigation into the pattern of use of complementary and alternative medicine (CAM) therapies that have been associated with oxidative stress pathways and other benefits among children with developmental disabilities may be an important first step. This is particularly imperative given the lack of evidence associated with the effectiveness of these CAM therapies and the potentially dangerous side effects [16].

Approximately 59 million Americans spend $30.2 billion on CAM and CAM practitioners annually [17]. Specifically, CAM utilization within the past 12 months among children under the age of 18 in the general population has been reported to be approximately 11.8%. This includes biologically based therapies (excluding vitamin/mineral use), mind-body therapies, alternative medicine systems, energy healing, and manipulative and body-based therapies [18]. However, CAM use among individuals with DS has been reported to be as high as 87%, 50% among individuals with ASD, and 56% among individuals with CP [16]. CAM includes dietary supplements, as well as nonnutritional modalities such as homeopathy and acupuncture. Many of the vitamins/supplements and therapies in CAM may act, ultimately, by decreasing oxidative stress and thus require further investigation to increase our understanding in the utilization of these therapies among children with developmental disabilities and aid in elucidating the etiology of these conditions [19, 20].

A growing number of families with children diagnosed with developmental disabilities, particularly ASD, are implementing CAM supplements to treat symptoms that they believe are affected by oxidative stress pathways [21, 22]. The reported use of CAM in this population ranges from 32 to 87% in the United States [23, 24]. Additionally, families of children with developmental disabilities have reported dissatisfaction with the services available to them to support their children and report higher levels of interest and use of CAM therapies [25]. Many families report that CAM offers an additional avenue of treatment other than conventional medical care and offer a sense of hope and control over their child's treatment, especially for children that have multiple cooccurring conditions that traditional therapies do not address and are associated with significant child and family stress [16, 26, 27].

The role of nutritional supplements and antioxidants such as vitamins, minerals, herbal, and nonherbal supplements in the reduction of oxidative stress levels has been extensively examined among different medical conditions including diabetes, heart disease, obesity, neurodegenerative diseases, and cancer [28–30]. However, this association is just beginning to be examined among children with developmental disabilities. Given the emerging evidence of a relationship between oxidative stress and the ontology of these conditions, it has been proposed that nutritional supplements and antioxidants, specifically, may be effective in reducing oxidative stress levels and hence be a potential avenue for prevention and/or intervention of symptoms seen in developmental disabilities. These CAM therapies may also demonstrate additional benefits of reducing symptomology and improving functionality by interacting with other symptoms, and thus it is imperative to understand patterns of use in this population to inform practice and further research [16].

Preliminary investigations in the field have begun to examine the prevalence of the use of these specific CAM therapies among children with developmental disabilities. However, it remains unclear whether specific CAM therapies (i) are effective at modulating symptoms or (ii) act via ameliorating oxidative stress. These questions, together with work to determine the safety of using these treatments, need to be answered and are of the utmost importance to aid medical providers in understanding CAM use among children with developmental disabilities. Informing on these questions will address whether any of these therapies may serve as a potential intervention avenue. The present study begins to address this gap by identifying CAM therapies families of children with these disabilities are likely to utilize, ensuring that providers can increase familiarity with such therapies to adequately inform their patients and to ensure that future research prioritize effects of these CAM therapies on specific developmental disabilities (ASD, CP, DS, ID, and DD) in relation to various pathways.

## 2. Methods

### 2.1. Participants and Procedure

Data from 10,218 participants between the ages of 4-17 included in the 2012 Child Complementary and Alternative Medicine (CAM) Supplement of the NHIS (National Health Interview Survey) were analyzed. The NHIS is a cross-sectional annual national representative in-person household survey providing information regarding data on health and healthcare utilization of the civilian noninstitutionalized child and adult population in the United States. The NHIS is conducted in the homes of participants using a computer-assisted personal interview, with telephone interviewing for follow-up if necessary. The Sample Child Core collects information about one randomly selected child aged 0-17 (the sample child) in each household. The NHIS uses a complex multistage sample design and survey weights are applied to examine estimates that are representative of the United States population. The Child CAM supplement was developed by the National Center for Health Statistics (NCHS) and the National Center for Complementary and Alternative Medicine to collect information about 34 alternative nonconventional health services, products, and practices commonly used in the United States by children aged 4-17. Further information about NHIS and the CAM supplement are available online (NCHS, 2012). We extracted variables related to CAM use, disability status, and sociodemographic background information. Due to all data being publicly available and the application of secondary data analyses, this study was deemed exempt by the institutional review board at <blinded for review>.

### 2.2. Measures

#### 2.2.1. CAM Use

The 2012 Child CAM supplement asked the proxy respondent if the sample child utilized particular CAM modalities. Thirty-four (34) CAM modalities were included in the supplement, but for the purpose of this study, only specific modalities (e.g., chelation, use of vitamin or minerals, use of herbal/nonvitamin supplements, and use of combination herb pills) were analyzed due to the relation of the particular modalities and oxidative stress. Proxy respondents were asked about the sample child's use as follows: “Has (sample child) ever used chelation therapy for his/her health? If they responded yes, they were asked, “if sample child has ever seen a provider or practitioner for chelation therapy?”, and asked, “if, during the past 12 months, sample child saw a practitioner for chelation therapy.” These questions were combined to assess if the sample child ever used chelation therapy. Proxy respondents were also asked if sample child has ever taken: (1) multivitamins or multiminerals; (2) vitamin A, B, C, D, E, H, or K, other than in a multivitamin or mineral; and/or (3) calcium, magnesium, iron, chromium, zinc, selenium, or potassium. They were also asked if sample child has ever taken any herbal or nonvitamin supplements listed: (1) combination herb pill, (2) açaí pills or gel caps, (3) pollen and bee products, (4) chondroitin, (5) coenzyme Q10 (CoQ10), (6) cranberry pills or capsules, (7) ginseng, (8) glucosamine, (9) green tea pills or epigallocatechin gallate (EGCG) pills, (10) melatonin, (11) milk thistle (silymarin), (12) MSM (methylsulfonylmethane), (13) digestive enzymes (Lactaid), (14) echinacea, (15) fish oil or omega 3 or DHA fatty acid or EPA, (16) garlic supplements, (17) ginkgo biloba, (18) probiotics or prebiotics, (19) SAM-e, (20) saw palmetto, or (21) valerian. They were also asked which supplements sample child has taken during the past 12 months.

#### 2.2.2. Sociodemographic Variables and Disability Status

Proxies reported on the sample child's sociodemographic variables. Income levels were determined based on federal poverty level status, which are based on the ratio of the family's income in the previous calendar year to the appropriate poverty threshold (given the family's size and number of dependents) defined by the U.S. Census Bureau for that year (Child Complementary and Alternative Medicine Supplement, 2012). Proxies reported on the highest level of education of any adult in the family. Proxies were also asked about the child's health insurance status at the time of the survey. Children were defined as uninsured if the child did not have any private health insurance, Medicare, Medicaid, CHIP, or state-sponsored or other government-sponsored health plans or military plan at the time of interview (Child Complementary and Alternative Medicine Supplement, 2012). Proxies were asked if a doctor or health professional ever told them that the sample child had (1) autism/autism spectrum disorder (ASD), (2) cerebral palsy (CP), (3) Down syndrome (DS), (4) intellectual disability (ID), or (5) any other developmental delay (DD).

### 2.3. Data Analysis Plan

#### 2.3.1. Data Analyses

All analyses were conducted in SPSS V.22. We adjusted for the complex probability survey design using sample weights to provide nationally representative estimates of the U.S. childhood population. Chi-square tests were conducted to compare the prevalence rates of CAM use among children with a particular disability (e.g., ASD, CP, ID, or DD) and those children without a history of the disability. Hierarchical logistic regression analyses were conducted to predict CAM use by disability type while controlling for relevant sociodemographic covariates (e.g., age, sex, race, family income, and caregiver education level).

## 3. Results

### 3.1. Preliminary Analyses

#### 3.1.1. Demographic and Disability Status Overview of the Sample

Demographic and disability status are reported in Table 1 for children with ASD, DS, CP, ID, and DD and all other children. Table 1 reports weighted percentages and mean group-differences by disability type (e.g., ASD, DS, CP, ID, and DD). Weighted data uses sample weighting in order to provide nationally representative estimates of the US childhood population. All disability groups were more likely to be male. For example, approximately 84.1% of youth with ASD were male and 50.6% of youth without ASD were male. Groups also differed with respect to age, ethnicity/race, geographic region, parental education, and family income.

### 3.2. Primary Analyses

**Figure 1:**
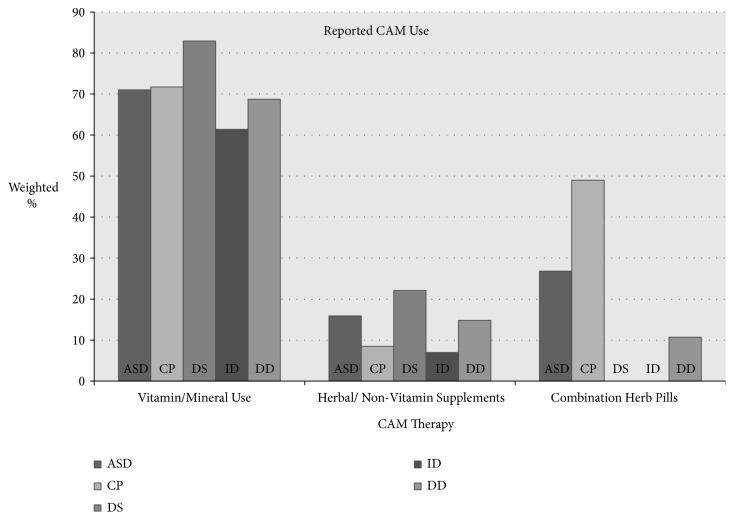
CAM use by diagnostic status. Note: ASD= autism spectrum disorders; CP= cerebral palsy; DS= Down syndrome; ID= intellectual disability; DD= developmental delay.

CAM use is reported in Table 2 and Figure 1 for the total sample and by disability group. Chi-square tests of independence with sample weights were calculated comparing the frequency of particular CAM use in those with a disability (i.e., ASD, CP, DS, ID, and DD) and those without the particular disability. For example, approximately 3% percent of youth with ASD were reported to utilize chelation therapy compared to approximately less than 1% of youth without ASD.

#### 3.2.1. CAM Use in ASD

The relation between ASD status and chelation use and vitamin/mineral use was statistically significant (*χ*^2^(1,*N*=56,499,231)=650,278.33,* p*<.001; *χ*^2^(1,*N*=56,360,315)=11,218.02,* p*<.001, respectively; Table 2). The relation between ASD status and the use of herbal or nonvitamin supplements and combination herb pills was also statistically significant (*χ*^2^(1,* N*=56,335,026)=115,595.22,* p*<.001; *χ*^2^(1,* N*=2,723,040)=75,700.86,* p*<.001, respectively). Youth with ASD were reported to be more likely to use chelation therapy, take vitamins or minerals, and use herbal or nonvitamin supplements and combination herb pills compared to those without ASD. Additionally, youth with ASD were reported more likely to use digestive enzymes, fish oil supplements, glucosamine, green tea, or EGCG pills, melatonin and probiotics compared to those without ASD.

#### 3.2.2. CAM Use in CP

The relation between CP status and chelation use and vitamin/mineral use was statistically significant (*χ*^2^(1,*N*=56,499,231)= 165.66,* p*<.001; *χ*^2^(1,*N*=56,360,315)=2,952.20,* p*<.001, respectively; Table 2). Additionally, the relation between CP status and use of herbal or nonvitamin supplements and combination herb pills was statistically significant (*χ*^2^(1,* N*=56,335,026)=1,139.31,* p*<.001; *χ*^2^(1,* N*=2,723,040)=64,671.93,* p*<.001, respectively). Youth with CP were reported to be less likely to use chelation therapy and take vitamin/minerals than those without CP. Also, youth with CP were reported to be more likely to use magnesium, iron, chromium, zinc, selenium or potassium, herbal or nonvitamin supplements, combination herb pills, and echinacea compared to those without CP.

#### 3.2.3. CAM Use in DS

The relationship between DS status and chelation use, vitamin/mineral use, use of herbal or nonvitamin supplements, and combination herb pills was statistically significant (*χ*^2^(1,* N*=56,499,231)= 67.52,* p*<.001; *χ*^2^(1,*N*=56,360,315)= 9,102.79,* p*<.001; *χ*^2^(1,* N*=56,335,026)= 27,203.45,* p*<.001; *χ*^2^(1,* N*=2,723,040)=661.28,* p*<.001, respectively). Refer to Table 2 for more information. Youth with DS were reported to be less likely to use chelation therapy and combination herb pills compared to those without DS. In addition, youth with DS were reported to be more likely to use vitamin/minerals, herbal or nonvitamin supplements, and probiotics and prebiotics than those without DS.

#### 3.2.4. CAM Use in ID

The relation between ID status and chelation use, vitamin/mineral use, herbal or nonvitamin supplements, and combination herb pills was statistically significant (*χ*^2^(1,*N*=56,500,291)=13,309.40,* p*<.001; *χ*^2^(1,*N*=56,373,119)=4,556.20,* p*<.001; *χ*^2^(1,* N*=56,347,830)=395.17,* p*<.001; *χ*^2^(1,* N*=2,723,040)=1,680.23,* p*<.001, respectively; Table 2). Youth with ID were reported to be more likely to use chelation therapy, vitamin A, B, C, D, E, H, or K, magnesium, iron, chromium, zinc, selenium, or potassium compared to those without ID. Youth with ID were also reported to be more likely to use herbal or nonvitamin supplements, fish oil supplements, garlic supplements, melatonin, probiotics, and prebiotics compared to those without ID. Additionally, youth with ID were reported to be less likely to use vitamin/minerals and combination herb pills compared to those without ID.

#### 3.2.5. CAM Use in DD

The relation between DD status and chelation use and vitamin/mineral use was statistically significant (*χ*^2^(1,*N*=56,459,516)=152,022.46,* p*<.001; *χ*^2^(1,*N*=56,332,344)=15,901.63,* p*<.001, respectively; Table 2). In addition, the relation between DD status and herbal or nonvitamin supplements and combination herb pill use was statistically significant (*χ*^2^(1,* N*=56,307,055)=343,516.98,* p*<.001; *χ*^2^(1,* N*=2,723,040)=38,908.99,* p*<.001, respectively). Youth with DD were reported to be more likely to use chelation therapy, vitamin/minerals, herbal and nonvitamin supplements, and combination herb pills than those without DD. Youth with DD were also more likely to use açaí pills, bee products cranberry pills and capsules, digestive enzymes, fish oil supplements, garlic supplements, green tea or EGCG pills, melatonin, milk thistle, MSM, and probiotics/prebiotics than those without DD.

### 3.3. Secondary Analyses

Hierarchical logistic regressions were conducted to control for the effect of covariates and determine odds ratios for disability group and CAM us. Covariates were entered on step 1 and disability types were entered on step 2.

#### 3.3.1. Chelation Use

Youth with ASD and youth with DD were significantly associated with an increase in the likelihood of using chelation therapy compared to those without ASD or without DD when controlling for covariates (Table 3). Additionally, youth with ID were significantly associated with a decrease in the likelihood of using chelation therapy compared to those without ID when controlling for covariates. CP and DS status were not significantly associated with chelation use (all* p*<.86).

#### 3.3.2. Vitamin/Mineral Use

Youth with ASD, CP, DS, and DD were significantly associated with an increase in the likelihood of using vitamin/mineral use compared to those without diagnoses when controlling for covariates (Table 3). Youth with ID were significantly associated with a decrease in the likelihood of vitamin/mineral use compared to those without ID when controlling for covariates.

#### 3.3.3. Vitamin A, B, C, D, E, H, or K Use

Youth with ASD, DS, ID, and DD were significantly associated with an increase in the likelihood of using specific vitamins compared to those without diagnoses when controlling for covariates (Table 3). CP status was significantly associated with a decrease in the likelihood of using specific vitamin use compared to those without CP when controlling for covariates

#### 3.3.4. Magnesium, Iron, Chromium, Zinc, Selenium, or Potassium Use

Youth with ASD, CP, DS, ID, and DD were significantly associated with an increase in the likelihood of using magnesium, iron, chromium, zinc, selenium, or potassium compared to youth without diagnoses when controlling for covariates (Table 3).

#### 3.3.5. Herbal or Nonvitamin Supplement Use

Youth with ASD, DS, and DD were significantly associated with an increase in the likelihood of using herbal or nonvitamin supplements compared to those without diagnoses (Table 3). Also, youth with CP and ID were significantly associated with a decrease in the likelihood of using herbal or nonvitamin supplements compared to those without CP or ID.

#### 3.3.6. Combination Herb Pill Use

Youth with ASD, CP, and DD were significantly associated with an increase in the likelihood of using combination herb pills compared to those without ASD CP or DD (Table 3). ID and DS status were not significantly associated with combination herb pill use (all* p*<.92).

## 4. Discussion

Developmental disabilities are increasing in prevalence and are typically associated with functional limitations and life-long support, highlighting the importance of examining potential prevention and intervention avenues. Although the etiology of these conditions is poorly understood, one emerging body of literature implicates the role of oxidative stress in the development and maintenance of these conditions, which may serve as a potential treatment target. As such, a growing number of families of children with developmental disabilities are utilizing CAM therapies, such as nutritional supplements and antioxidants that are proposed to interact with oxidative stress pathways [16, 28–30].

This study examined the utilization of CAM therapies associated with a potential to ameliorate oxidative stress in a large population-based sample of children with developmental disabilities and typically developing comparison children. Findings of this study support previous research that children with developmental disabilities are more likely to use CAM therapies compared to neurotypical youth [16]. Additionally, this study provides preliminary evidence for the use of CAM therapies by disability type associated with a potential to ameliorate oxidative stress deficiencies among children with developmental disabilities, providing a promising arena for future research. While previous studies have examined CAM therapy use among specific disability groups, this is the first study to examine CAM therapy use in relation to oxidative stress among multiple developmental disabilities in a large representative sample. Specifically, children with an ASD diagnosis were more likely to utilize the most number of CAM therapies compared to children with ID, DD, DS, and CP. Children diagnosed with DD demonstrated similar utilization rates to children diagnosed with ASD, which may suggest parents of children with ASD and DD are seeking alternative treatment options to alleviate impairment [16]. Additionally, children with a CP diagnosis were more likely to use specific dietary supplements and less likely to use other forms of CAM therapy, and children with a DS diagnosis were more likely to use vitamins and minerals compared to other CAM therapies. This may suggest that parents of children with CP or DS are more exclusive in the types of CAM that they utilize for their children. Finally, children with an ID diagnosis were more likely to use chelation therapy, specific vitamins and minerals and herbal and nonherbal supplements compared to those without an ID diagnosis and less likely to use other forms of CAM therapy. Overall, results suggest that children with developmental disabilities utilize CAM therapies that may interact with oxidative stress pathways more frequently than children without developmental disabilities, and the utilization of these therapies varies by disability type.

These findings are essential for medical providers. Given that we do not sufficiently know the impact of CAM products on improving disability-related symptoms or alleviating oxidative stress, adverse events may, in fact, exceed potential benefits and thus negatively impact the overall well-being of children. At this time, the potential benefits of CAM therapies represented in the literature are mixed. For example, in controlled-randomized studies in children and adolescents with ASD, melatonin use was shown to be superior in treating sleep disturbances with no difference in adverse effects observed between placebo and treatment groups [31, 32]. In contrast, studies examining efficacy of omega-3 fatty acids have failed to demonstrate an improvement in core symptoms of hyperactivity, although supplementation was reported as well-tolerated [33–35]. Some CAM modalities such as chelation therapy have been widely discouraged due to serious events such as hypocalcemia, which can be fatal and which have been shown to have no significant impact in symptoms of ASD [36, 37]. The use of multivitamins and micronutrients has also been cautioned in a study by Stewart et al. [38] until appropriate dosing knowledge is available, in order to minimize adverse effects from excessive intake. Given this, the authors recommend that physicians counsel families with respect to its use [38].

Despite the support by insurance companies to cover costs of prescription medication for the treatment of core ASD symptomology, these treatments have also reported adverse events. In a systematic review [39], risperidone and aripiprazole for ASD symptomology were shown to be beneficial. However, significant adverse events were also associated with their use. The review recommended that families be advised to use these prescriptions, only in cases of heightened risk of injury or severe impairment of the child. All other medical interventions such as citalopram, fluoxetine, and sertraline were found to lack sufficient evidence to support recommendation on the basis of benefits exceeding adverse events [39].

The increasing utilization and willingness of families to pay out-of-pocket for CAM therapies may be explained given the mixed and indefinite state of evidence for both CAM and prescription medication for the treatment of developmental disabilities symptomology. It is, as a result, imperative that medical providers have a greater understanding of CAM product use in children within specific disability groups. Medical providers should ask direct questions related to their use, since most parents hesitate in reporting the use of CAM products to their medical provider [16]. This can lower the potential for adverse reactions and outcomes for the child, particularly as many of these supplements are unregulated, and may adversely interact with conventional medicines or be accompanied by unfound health benefit claims. The results of this study provide encouragement for health workers to enhance their awareness and familiarity with frequently used CAM therapies to adequately inform and guide utilization among their patients and families.

This study, however, has its limitations. The NHIS survey data were collected using a cross-sectional design and parent report of a child, which does not indicate changes in types and frequency of CAM utilization. A longitudinal study would alleviate this limitation by allowing children to respond as adults in the future, as well as determine patterns of usage over time. It may also illustrate differences in CAM utilization across the lifespan which would be valuable for future research. In addition, the sample size of individuals with DS is small (n=12), and thus generalizability of these findings for this group is greatly limited. Other limitations include the lack of clarity on the purpose of CAM utilization and the families impressions of the overall benefit or negative outcomes associated with CAM use. No question(s) explicitly addressed these on the national survey. It would be beneficial for future surveys to collect such pertinent information, to help understand the rationale for CAM use, and to examine and explain the reported observed effects of CAM. Additionally, the question on the national survey assessing specific vitamin use inquires about “vitamin H,” which is commonly referred to as biotin [40]. This may have resulted in under-reporting of use, and consequently actual use may be misrepresented in the data. Future studies should include clarification of commonly referred nomenclature, examine the intended purpose of utilizing particular CAM therapies, and investigate the positive or adverse effects of CAM utilization on specific disability groups and as such identify optimal dosage for maximizing reported benefits and minimizing adverse effects.

## 5. Conclusions

This study provides preliminary evidence into the utilization of CAM therapies associated with oxidative stress among children with developmental disabilities. As parents continue to use supplements to alleviate the symptoms associated with these disabilities, there is a clear need for robust placebo-controlled clinical trials to evaluate the safety, efficacy and optimal dosage of the most popular CAM therapies identified in this study. Furthermore, understanding the link between these therapies and their potential modulation of oxidative stress will be essential for clinicians and families to make more informed decisions regarding CAM modalities in individuals with disabilities and may serve as a potential avenue for intervention efforts once additional research is conducted [4].

## Figures and Tables

**Table 1:** Sociodemographic characteristics of total population and by disability groups.

Variable	Total Sample (n= 10,218) Weighted %	Weighted %		*p*	Weighted %		*p*	Weighted %		*p*	Weighted %		*p*	Weighted %		*p*
		ASD (n= 144)	Non-ASD (n= 10,067)		CP (n= 33)	Non-CP (n= 10, 178)		DS (n=12)	Non-DS (n=10,199)		ID (n= 129)	Non-ID (n=10,085)		DD (n= 487)	Non-DD (n= 9,719)	
Estimated Proportion	100	1.4	98.6	-* *-	.3	99.7	-* *-	.1	99.9	-* *-	1.2	98.8	-* *-	4.9	95.1	-* *-
Age, M (SD)	10.5(4.03)	10.3(3.66)	10.5(4.03)	<.001	10.5 (3.64)	10.5 (4.03)	<.001	10.08 (4.08)	10.53 (4.03)	<.001	11.74 (3.57)	10.51 (4.03)	<.001	10.33 (4.0)	10.53 (4.03)	<.001
%Male	51.1	84.1	50.6	<.001	64.9	51.1	<.001	62.6	51.1	<.001	73.6	50.8	<.001	67.8	50.2	<.001
Hispanic	23.5	15.7	23.6	<.001	17.5	23.5	<.001	14.8	23.5	<.001	21	23.5	<.001	16.1	23.9	<.001
White, Non-Hispanic	53.7	56.7	53.7	<.001	53.6	53.7	<.001	79.3	53.7	<.001	51.4	53.7	<.001	62.9	53.2	<.001
Black, Non-Hispanic	13.4	15.3	13.4	<.001	21.8	13.4	<.001	0	13.4	<.001	20	13.3	<.001	12.9	13.4	<.001
Multiple, Non-Hispanic	9.4	12.3	9.4	<.001	7	9.4	<.001	6	9.4	<.001	7.6	9.5	<.001	8.1	9.5	<.001
Geographic Region																
Mid-West	22.7	23	22.7	<.001	17	22.7	<.001	37.3	22.7	<.001	21.8	22.7	<.001	21.2	22.7	<.001
North-East	16.7	14.2	16.7	<.001	13.9	16.7	<.001	6	16.7	<.001	12.4	16.7	<.001	18.4	16.6	<.001
South	36.9	45.2	36.8	<.001	41.2	36.9	<.001	23.2	37	<.001	42.5	36.9	<.001	39.6	36.8	<.001
West	23.7	17.6	23.8	<.001	27.9	23.7	<.001	33.5	23.7	<.001	23.3	23.7	<.001	20.8	23.9	<.001
Parental Education																
Less than High School	10.6	5.1	10.6	<.001	3.5	10.6	<.001	7.2	10.6	<.001	13.9	10.5	<.001	10.9	10.5	<.001
HS Diploma or GED	18.9	13.1	19	<.001	31.5	18.8	<.001	6.2	18.9	<.001	22.2	18.8	<.001	17.1	18.9	<.001
Some College or Higher	70.6	81.8	70.4	<.001	65	70.6	<.001	86.6	70.5	<.001	63.9	70.7	<.001	72	70.5	<.001
% Insured	7	93	93	.88	96.2	93	<.001	98.2	93	<.001	97.6	92.9	<.001	93.6	93	<.001
Income Level Based on FPL																
0-99% FPL	21	18.5	21	<.001	8.6	21	<.001	13.7	21	<.001	23.8	20.9	<.001	24.8	20.8	<.001
100-199% FPL	23.9	30.6	23.8	<.001	32	23.9	<.001	8.3	23.9	<.001	28.7	23.8	<.001	24.6	23.8	<.001
200-399% FPL	28.9	31.4	28.9	<.001	33.6	28.9	<.001	67	28.9	<.001	33.2	28.9	<.001	25.8	29.1	<.001
400% FPL or Above	26.2	19.6	26.3	<.001	25.9	26.2	<.001	11	26.2	<.001	14.3	26.4	<.001	24.7	26.3	<.001

Note: all percentages are weighted to provide nationally representative estimates of the US childhood population. Sample sizes reported are not weighted.

ASD= autism spectrum disorders; CP= cerebral palsy; DS= Down syndrome; ID= intellectual disability; DD=developmental delay; HS=high school; FPL= federal poverty level; Ca=calcium; Mg=magnesium; FE=iron; Zn=zinc; Se=selenium; K=potassium.

^1^Percent that had seizures in the past 12 months.

**Table 2:** CAM use of total population and by disability groups.

Variable	Total Sample Weighted %	Weighted %		*p*	Weighted %		*p*	Weighted %		*p*	Weighted %		*p*	Weighted %		*p*
		ASD	Non-ASD		CP	Non-CP		DS	Non-DS		ID	Non-ID		DD	Non-DD	
Chelation Use	0.1	3	0.1	<.001	0	0.1	<.001	0	.1	<.001	0.5	0.1	<.001	0.8	0.1	<.001
Vitamin/Mineral Use	65.2	71	65.2	<.001	71.7	65.2	<.001	82.9	65.2	<.001	61.4	65.3	<.001	68.7	65	<.001
Specific Vitamin Use^1^	13.3	17.9	13.2	<.001	11.3	13.3	<.001	41.5	13.3	<.001	19.4	13.2	<.001	16.2	13.1	<.001
Mg, Fe, Cr, Zn, Se, or K Use	7.6	15.2	7.5	<.001	15.2	7.6	<.001	13.4	7.6	<001	12.5	7.5	<.001	14.4	7.2	<.001
Herbal or Non-Vitamin Supplement Use	6.4	15.9	6.3	<.001	8.5	6.4	<.001	22.1	6.4	<.001	7	6.4	<.001	14.8	6	<.001
Combination Herb Pill Use^2^	4.3	26.8	3.8	<.001	49	4.1	<.001	0	4.3	<.001	0	4.4	<.001	10.7	3.4	<.001
Co-Enzyme Q10 Use^2^	1.4	0	1.4	<.001	0	1.4	<.001	0	1.4	<.001	0	1.4	<.001	1.3	1.4	.06
Açaí Pill Use^2^	2.8	0	2.9	<.001	0	2.8	<.001	0	2.8	<.001	0	2.8	<.001	5.3	2.4	<.001
Bee Product Use^2^	5.4	0	5.5	<.001	0	5.4	<.001	0	5.4	<.001	0	5.4	<.001	8.1	5	<.001
Chondroitin Use^2^	1.4	0	1.4	<.001	0	1.4	<.001	0	1.4	<.001	0	1.4	<.001	0	1.6	<.001
Cranberry Pills or Capsule Use^2^	3.3	0	3.4	<.001	0	3.3	<.001	0	3.3	<.001	0	3.4	<.001	5.6	3	<.001
Digestive Enzymes Use^2^	8.3	38.7	7.6	<.001	0	8.3	<.001	0	8.3	<.001	0	8.4	<.001	13.7	7.5	<.001
Echinacea Use^2^	19.6	0	20	<.001	49	19.5	<.001	0	19.7	<.001	0	19.9	<.001	8.2	21.3	<.001
Fish Oil Supplement Use^2^	43.6	69.2	43.1	<.001	0	43.9	<.001	0	43.9	<.001	49.9	43.6	<.001	46.3	43.2	<.001
Garlic Supplements Use^2^	5.5	0	5.7	<.001	0	5.6	<.001	0	5.6	<.001	7.9	5.5	<.001	7.6	5.3	<.001
Ginkgo Bilboa Use^2^	1.9	0	1.9	<.001	0	1.9	<.001	0	1.9	<.001	0	1.9	<.001	1.3	2	<.001
Ginseng Use^2^	4.0	0	4.1	<.001	0	4	<.001	0	4	<.001	0	4	<.001	3.3	4.1	<.001
Glucosamine Use^2^	3.1	5.1	3.1	<.001	0	3.1	<.001	0	3.1	<.001	0	3.2	<.001	0.9	3.4	<.001
Green Tea or EGCG Pill Use^2^	0.9	8.5	.7	<.001	0	0.9	<.001	0	0.9	<.001	0	0.9	<.001	1.5	0.8	<.001
Melatonin Use^2^	26.1	69.8	25.1	<.001	5.8	26.2	<.001	21.2	26.1	<.001	49.7	25.8	<.001	36.2	24.6	<.001
Milk Thistle Use^2^	0.9	0	1	<.001	0	1	<.001	0	1	<.001	0	1	<.001	3.3	0.6	<.001
MSM Use^2^	0.4	0	0.4	<.001	0	0.4	<.001	0	0.4	<.001	0	0.4	<.001	1.3	0.3	<.001
Probiotics or Prebiotics Use^2^	21.1	53.3	20.4	<.001	0	21.2	<.001	78.8	20.8	<.001	46	20.8	<.001	32.8	19.4	<.001
Sam-E Use^2^	0.6	0	0.6	<.001	0	0.6	<.001	0	0.6	<.001	0	0.6	<.001	0	0.7	<.001
Saw Palmetto Use^2^	0	0	0	-* *-	0	0	-* *-	0	0	-* *-	0	0	-* *-	0	0	-* *-
Valerian Use^2^	3.1	0	3.2	<.001	0	3.1	<.001	0	3.1	<.001	0	3.2	<.001	0	3.6	<.001

Mg=magnesium; Fe= iron; Cr=chromium; Zn=zinc; Se=selenium; K=potassium.

^1^Ever taken vitamin A, B, C, D, E, H, or K.

^2^Use in the past 12 months.

**Table 3:** Summary of hierarchical logistic regression analyses for variables predicting CAM use.

Variable	*β*	SE B	Wald	OR	95% CI OR
Chelation Use					
ASD*∗*	3.65	.02	56591.40	38.34	37.21-39.51
CP	-13.84	79.48	.03	.00	-* *-* *-* *-
DS	-15.78	119.64	.02	.00	-* *-* *-* *-
ID*∗*	-.30	.02	257.26	.74	.71-.77
DD*∗*	.67	.02	2060.05	1.94	1.89-2.00
Vitamin/Mineral Use					
ASD*∗*	.14	.003	2352.50	1.15	1.14-1.15
CP*∗*	.26	.006	1964.42	1.29	1.28-1.31
DS*∗*	.88	.01	6714.58	2.41	2.36-2.46
ID*∗*	-.19	.003	4716.94	.83	.82-.83
DD*∗*	.17	.002	12713.32	1.19	1.18-1.19
Specific Vitamin Use^1^					
ASD*∗*	.19	.003	2979.92	1.21	1.20-1.22
CP*∗*	-.29	.008	1274.06	.75	.74-.76
DS*∗*	1.38	.009	26233.91	3.99	3.93-4.06
ID*∗*	.11	.004	941.98	1.12	1.11-1.13
DD*∗*	.20	.002	11407.47	1.23	1.22-1.23
Mg, Fe, Cr, Zn, Se, or K Use					
ASD*∗*	.35	.004	8679.72	1.41	1.41-1.43
CP*∗*	.37	.007	2603.43	1.45	1.43-1.47
DS*∗*	.33	.01	733.54	1.39	1.35-1.42
ID*∗*	.08	.004	320.99	1.08	1.07-1.09
DD*∗*	.75	.002	130114.47	2.11	2.10-2.12
Herbal or Non-Vitamin Supplement Use					
ASD*∗*	.42	.004	12471.64	1.52	1.51-1.53
CP*∗*	-.10	.009	113.00	.91	.89-.92
DS*∗*	1.48	.01	19563.34	4.38	4.29-4.48
ID*∗*	-.77	.006	19245.58	.46	.46-.47
DD*∗*	.98	.002	222831.17	2.67	2.66-2.68
Combination Herb Pill Use					
ASD*∗*	1.74	.01	20711.72	5.73	5.60-5.87
CP*∗*	3.01	.02	25373.85	20.20	19.46-20.96
DS	-16.97	278.99	.004	.00	-* *-* *-* *-
ID	-17.97	195.43	.008	.00	-* *-* *-* *-
DD*∗*	1.07	.009	15119.70	2.91	2.86-2.95

*∗p*<.001; Mg=magnesium; Fe= iron; Cr= chromium; Zn=zinc; Se=selenium; K=potassium.

^1^Vitamin A, B, C, D, E, H, or K use.

## Data Availability

The Child Complementary and Alternative Medicine (CAM) supplement of the National Health Interview Survey (NHIS) is available by requesting the data from http://action.cahmi.org/help/dataset.
